# Quality of Life Is Not Deteriorated After Extrapleural Pneumonectomy vs. (Extended) Pleurectomy/Decortication in Patients With Malignant Pleural Mesothelioma

**DOI:** 10.3389/fsurg.2021.766033

**Published:** 2021-12-08

**Authors:** Olivia Lauk, Miriam Patella, Thomas Neuer, Ilhan Inci, Walter Weder, Isabelle Opitz

**Affiliations:** Department of Thoracic Surgery, University Hospital Zurich, Zurich, Switzerland

**Keywords:** malignant pleural mesothelioma, induction chemotherapy, quality of life, lung function, macroscopic complete resection, extrapleural pneumonectomy (EPP), extended pleurectomy/decortication

## Abstract

**Background:** Extrapleural pneumonectomy (EPP) for malignant pleural mesothelioma (MPM) is highly abandoned due to high morbidity and mortality rates and impaired quality of life (QoL). However, there are still rare indications for this intervention. The aim of this longitudinal prospective study was to monitor QoL and lung function in patients undergoing EPP and compare the outcomes with extended pleurectomy/decortication [(E)PD].

**Methods:** Between June 2013 and June 2017, 42 patients underwent induction chemotherapy followed by either EPP (*n* = 7) or (E)PD (*n* = 35). All patients filled out the EORTC QLC-C15-PAL, –LC13, and SF-36 self-rating questionnaires pre-operatively, 6 weeks and 4 months after the operation. Additionally, lung function was measured pre-operatively and 4 months post-operatively.

**Results:** We observed no significant differences in all QoL categories (general global health, pain, and dyspnea) between both surgical procedures, over the whole observation period. Moreover, a general tendency toward restoration of the pre-operative QoL status was documented at 4 months after the both operations. Forced expiratory volume in 1 s (FEV1) showed a significant decrease after surgery in both the groups [EPP group *p* = 0.06 and (E)PD group *p* < 0.001]; also, the forced volume vital capacity (FVC) significantly decreased (EPP group *p* = 0.046 P/D group <0.001). Diffusion capacity did not show significant changes.

**Conclusion:** According to these results, QoL is no longer severely impaired after EPP compared with EPD, and therefore should not be used as an argument against EPP in principle. However, indication has to be carefully evaluated for each patient.

## Background

Optimal radical treatment for malignant pleural mesothelioma (MPM) involves a multimodal approach, including induction chemotherapy followed by surgery (1, 2). A shift in the surgical approach for MPM from extrapleural pneumonectomy (EPP) to (extended) pleurectomy/decortication [(E)PD] has been observed in the recent past. Crucial for this shift was a rethinking after the mesothelioma and radical surgery (MARS) trial. Although the study was not designed to test the benefit of surgery, EPP was associated with higher mortality compared with chemotherapy alone (3). EPP was highly abandoned in the past because of higher reported morbidity and mortality rates, and deteriorating impact on the quality of life (QoL) in most patients (4–6). However, also, today indications for EPP do exist in selected patients; therefore, the aim of this analysis was to prospectively assess QoL and lung function in patients undergoing EPP and to compare the results to the (E)PD procedure, until 4 months post-operatively.

## Methods

This study of prospectively collected questionnaires was conducted between June 2013 and June 2017 and included 42 patients undergoing induction chemotherapy followed by either EPP (*n* = 7) or (E)PD (*n* = 35). All patients either underwent surgical or interventional biopsy via fine needle aspiration by endobronchial ultrasound (EBUS) or via thoracoscopic-guided pleural biopsy. Pre-operative mediastinal staging was performed either by EBUS or by mediastinoscopy depending on the size and the affected lymph nodes seen on pre-operative imaging. All patients passed our institutional multidisciplinary tumor board, and treatment allocation was based on therapy approach, including macroscopic complete resection (MCR) according to current guidelines (1), the patient's performance status and also the patients' wish. Decisions for EPP or EPD were made based on the patient's tumor stage and volume, intra-operative findings with regard to parenchymal infiltration, and fresh frozen sections. In more recent years, EPD became the preferred approach. Three different self-rating questionnaires (EORTC QLC-C15-PAL, –LC13, and SF-36) were submitted pre-operatively and post-operatively (6 weeks and 4 months after the operation) for the evaluation of patient's QoL. Additionally, pulmonary function test was performed pre-operatively and 4 months post-operatively (7–9).

As there are no specific questionnaires existing for patients suffering from pleural mesothelioma, we tried to integrate lung specific and palliative questions on top of the core questionnaire SF-36. This reflects the best this patient cohort where lung is affected and also that this disease is life-limiting.

The two surgical procedures [(E)PD and EPP] were performed as described previously in our institution (10, 11).

Local ethics committee approval was given for the analysis of the mesothelioma database (StV 29-2009, EK-ZH 2012-0094). All patients signed their written informed consent.

### Quality of Life Assessment

Three self-rating surveys were used to assess QoL: The German versions of the European Organization for Research and Treatment of Cancer (EORTC) QoL questionnaires *QLQ-C15-PAL, QLQ-LC13*, and the core Short Form *(SF)-36* questionnaire. Areas covered by the SF-36 derived from a larger 149 item instrument including eight sections (vitality, physical functioning, bodily pain, general health perceptions, physical role functioning, emotional role functioning and social role functioning, and mental health). With the *QLQ-LC15-PAL* questionnaire, only single items are assessed (pain, physical function, emotional function, fatigue, global health status/quality of life, nausea/vomiting, appetite, dyspnea, constipation, and sleep).

The *QLQ-LC13* questionnaire assesses one multi-item scale (dyspnea scale) and a series of single items (pain, coughing, sore mouth, dysphagia, peripheral neuropathy, alopecia, and hemoptysis). These can be categorized in lung cancer-associated symptoms (i.e., coughing, hemoptysis, dyspnea, and pain) and side effects from conventional chemo- and radiotherapy (i.e., hair loss, neuropathy, sore mouth, and dysphagia).

The dyspnea scale consists of three items (dyspnea when resting, dyspnea when walking, and dyspnea when climbing stairs). This scale was only calculated if all three items of this multi-item scale have been answered.

In general, a higher score for symptom scales presents a higher level of symptomatology. For global health/QoL and functional scales, higher scores represent an improvement (7). All questionnaires were handed out by the nurse and reviewed by the treating surgeon in the clinic, and any uncertainties were discussed with the patient.

### Lung Function Assessment

Pulmonary function test was performed pre-operatively and 4 months post-operatively. The following pulmonary parameters were analyzed: forced expiratory volume in 1 s (FEV1), forced vital capacity (FVC), and diffusing capacity of the lung for carbon monoxide (DLCO), and displayed as percentages of predicted normality.

### Statistical Analysis

For statistical analysis, R-software version 3.6.2 was used. A *p*-value of <0.05 was considered as statistically significant. This study is a qualitative study without quantitative statement. In accordance with the procedures recommended by the EORTC, scores were linearly converted to a scale ranging from 0 to 100 for each patient. For the global health/QoL and functional scales, higher scores represent a higher level of functioning. Furthermore, for the symptom scales, higher scores represent a greater symptom burden. Results were reported as median and interquartile range (IQR). Paired Wilcoxon-signed rank test was used to compare paired samples between two different time points (pre-surgery vs. 6 weeks post-surgery, pre-surgery vs. 4 months post-surgery, and 6 weeks vs. 4 months post-surgery).

A difference-in-differences analysis was performed to reduce confounding influence. With this method, one takes the difference between the values of every patient at 4 months after the operation and pre-operatively. Further, these differences were compared between the two surgical groups. All variables are indicated as mean of the differences with the standard deviation for each group, and *p*-values were calculated with the linear regression of the corresponding Mann–Whitney test and *t*-test.

Differences in the pulmonary function test according to the surgical procedure and post-operative time point (6 weeks and 4 months) were calculated using the non-parametric Wilcoxon–Mann–Whitney test. A *p*-value of <0.05 was statistically significant.

We also assessed the peri- and post-operative morbidity and mortality rate. Major morbidity was defined as complications necessitating reoperation, chylothorax, patch failure, empyema, bronchopleural fistula, thromboembolic events, persistent air leak, and acute respiratory distress syndrome (ARDS).

Overall survival (OS) and progression-free survival (PFS) were calculated from the date of diagnosis until death or last follow-up and until the date of first recurrence of the last follow-up or death. Only complete cases were considered. Follow-up was performed with computed tomography (CT) and positron emission tomography/CT (PET/CT) scans in an alternating manner according to our institutional guidelines.

## Results

**Figure 1:**
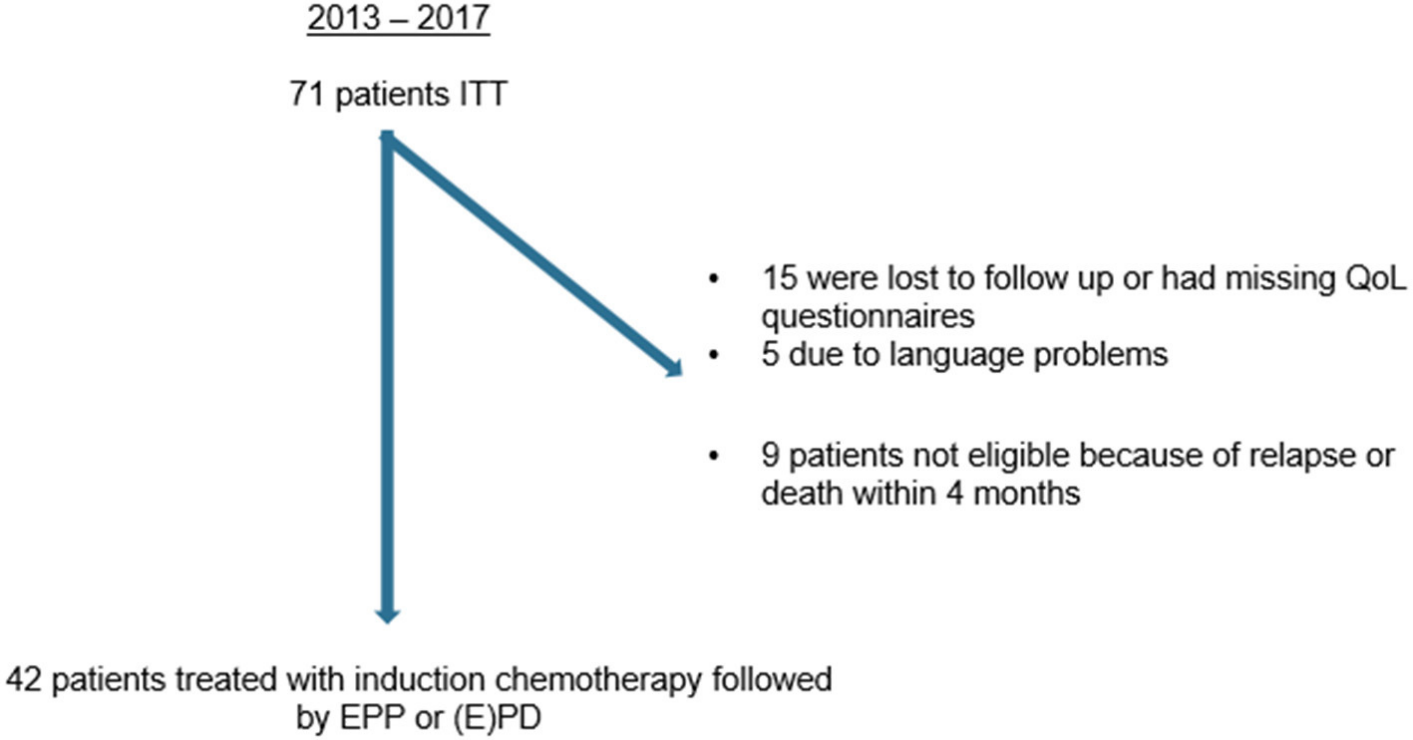
Flow chart. ITT, indented to be treated.

Between 2013 and 2017, 42 out of 71 patients with MPM undergoing MCR after induction chemotherapy were eligible for inclusion (Figure 1). Twenty -nine patients were excluded due to the following reasons: early relapse/death (*n* = 5/*n* = 4), lost at follow-up (FU) or missing QoL questionnaire pre-operatively [FU (*n* = 15)], and language problems (*n* = 5). Forty-two patients treated within a multimodality therapy approach followed by either EPP (*n* = 7) or EPD (*n* = 35). Nineteen patients out of 42 were additionally treated within our INFLuenCe Meso trial I (*n* = 5) and trial II (*n* = 14; NCT01644994) (intracavitary cisplatin-fibrin localized chemotherapy after pleurectomy/decortication or extrapleural pneumonectomy for the treatment of patients with malignant pleural mesothelioma) (12). This clinical study evaluated the safety and efficacy of intracavitary use of cisplatin-fibrin after MCR.

Demographic, surgical, and clinical data are listed in Table 1. The median age at surgery was 65 years and the majority had a good performance status (PS 0–1, 86%). Overall 76% of the patients had epithelioid histotype and a pathological IMIG stage I–II (67%) according to the eighth edition of the IMIG classification. Eleven patients had advanced tumor stage (IMIG stage IV: 26%).

**Table 1:** Patient's characteristics and demographics.

**Covariate**	**Overall**	**EPD**	**EPP**	***p***
***n***	**42 median [range]**	**35 median [range]**	**7 median [range]**	
Age at surgery (years)	65.04 [40.69–77]	65 [40.69–77]	67.6 [49.54–74]	0.51
Smoking	16 (39%)	14 (41.2%)	2 (28.6%)	0.61
Weight loss	12 (43.3%)	9 (31%)	3 (50%)	0.68
Category (%)				0.29
EPD	35 (83.3)	35 (83.3)	0 (0.0)	
EPP	7 (16.7)	0 (0.0)	7 (100.0)	
Length of stay	19 [8–40]	20 [8–40]	16 [13–19]	0.05
ECOG PS <1	25 (78%)	22 (82%)	3 (60%)	0.06
Follow-up status	19 (46%)	14 (41%)	5 (71%)	0.3
Epithelioid histotype (surgery)	32 (76%)	27 (77%)	5 (71%)	0.81
Intensive care unit stay	4 [1–21]	1 [1–21]	3 [1–6]	0.66
Right-sided laterality	18 (43%)	13 (37%)	5 (71%)	0.77
ASA classification				0.06
1	1 (2%)	1 (3%)	0	
2	22 (52%)	21 (60%)	1 (14%)	
3	19 (45%)	13 (37%)	6 (86%)	
Surgery duration (mins)	552.5 [255–850]	604 [316–850]	350 [255–435]	**<0.001**
IMIG 8th classification				0.42
1	5 (12%)	5 (14%)	0	
2	23 (55%)	20 (57%)	3 (43%)	
3	1 (2%)	1 (3%)	0	
4	11 (26%)	8 (23%)	3 (43%)	
Unknown	2 (5%)	1 (3%)	1 (14%)	
RECIST (%)				0.3
PD	6 (14%)	5 (14%)	1 (14%)	
PR	11 (26%)	10 (29%)	1 (14%)	
SD	21 (50%)	18 (51%)	3 (43%)	
Unknown	4 (10%)	2 (6%)	2 (29%)	
Major morbidity (%)	11 (28.2) [NA's: 3 (7%)]	8 (24.2) [NA's: 2 (6%)]	3 (50.0) [NA's: 1 (3%)]	0.43
30-day mortality (%)	0 [NA's: 2 (5%)]	0 [NA's: 0 (0%)]	0 [NA's: 2 (6%)]	**<0.001**
90-day mortality (%)	0 [NA's: 2 (5%)]	0 [NA's: 0 (0%)]	0 [NA's: 2 (29%)]	**<0.001**

*Results are expressed as median [range] unless otherwise specified NA, not available; all NAs have been excluded in the calculation; PD, progressive disease; PR, partial response; SD, stable disease*.

The median overall survival for the EPP group was 21 months (95% CI: 0.12-NA), and patients undergoing (E)PD had a median overall survival of 33 months (95% CI: 23-NA). The median PFS for the EPP group was 11 months (95% CI: 10-NA) and for the (E)PD group 13 months (95% CI: 11–15).

Comparison of the two different surgical approaches [(E)PD vs. EPP] showed a significant difference only in the median surgery duration (604 vs. 344 min, *p* < 0.01). The overall 30- /90-day mortality for both procedures was 0%.

Each of the three questionnaires was evaluated separately. No significant difference in quality of life of patients with (E)PD compared with patients with EPP was observed at 6 weeks and 4 months post-operatively.

**Figure 2:**
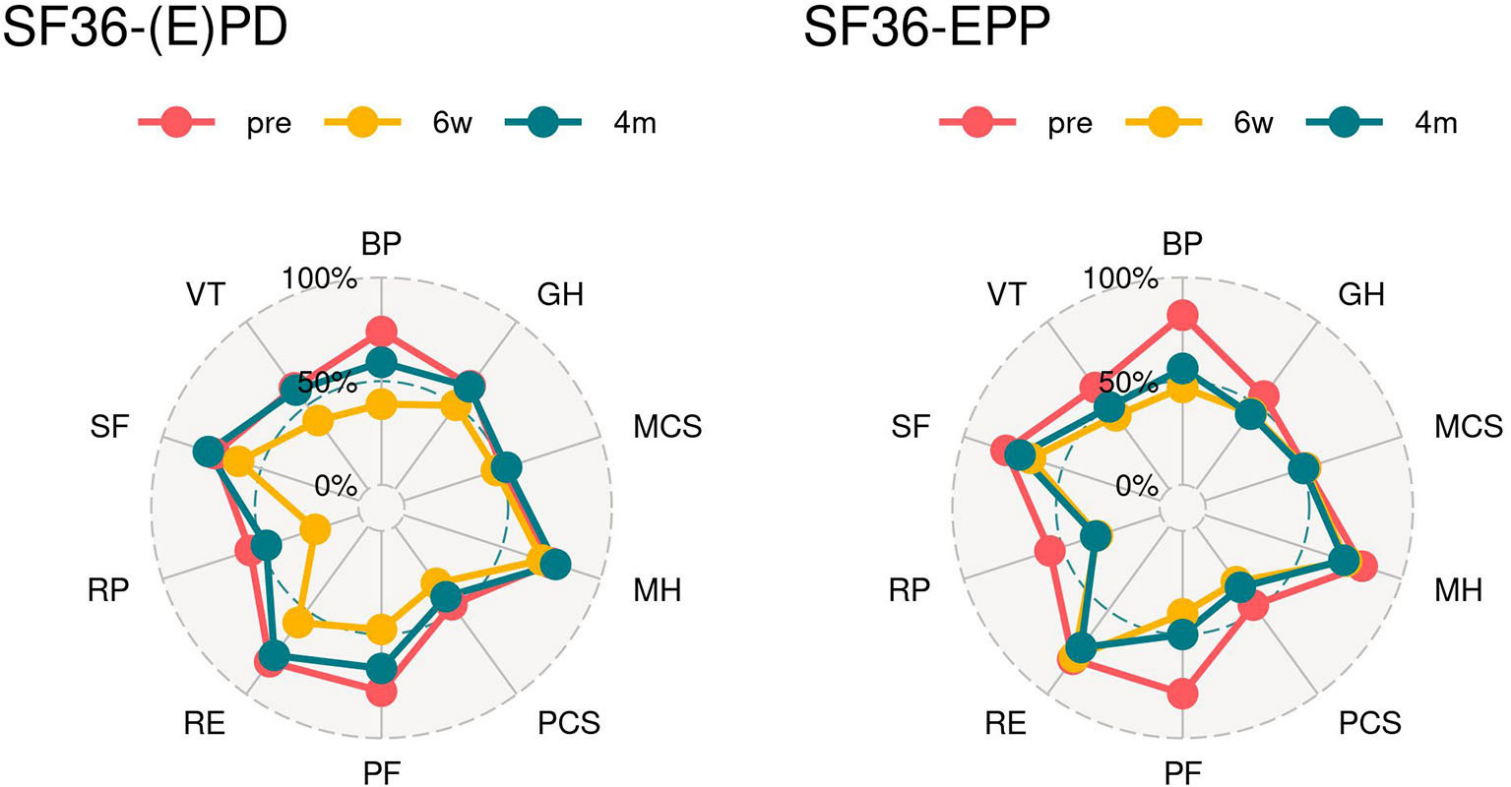
Spider graphs. SF 36 (1986): score of zero is equivalent to maximum disability and a score of 100 is equivalent to no disability. PF, physical functioning; RP, role physical; BP, bodily pain; GH, global health; VT, vitality; SF, social functioning; RE, role emotional; MH, mental health; PCS, physical component summary; MCS, mental component summary.

In the SF 36 questionnaire, there was no significant differences in each scale between both groups. However, in the (E)PD group mental component status (MCS) and mental health (MH) showed already an improvement compared to baseline values at 6 weeks post-operatively. In the EPP group, MCS, MH, and role emotional (RE) were already back to or lower than baseline values at 6 weeks post-operatively and 4 months post-operatively; baseline values have been reached by almost every item in the (E)PD group except for bodily pain (BP), physical functioning (PF), and role physical (RP). Whereas, in the EPP group, a return partly overshooting to baseline values was seen for BP, global health (GH), MH, physical component status (PCS), PF, RP, and vitality (VT) (Table 2 and Figure 2).

**Table 2:** SF 36 (1986).

**Attribute**	**Pre**	**6 w**	**4 m**	***p* (6 w-pre)**	***p* (4 m-pre)**	***p* (4 m−6 w)**	**Pre**	**6 w**	**4 m**	***p* (6 w-pre)**	***p* (4 m-pre)**	***p* (4 m−6 w)**
	**EPD**						**EPP**					
BP	74 (52–100)	41 (22–52)	57 (41–74)	**0**	**0.01**	**0**	84 (77–92)	51 (31–52)	52 (46–74)	**0.04**	0.11	0.58
GH	67 (48–72)	47 (41–62)	62 (56–72)	**0**	0.69	**0**	56 (46–64)	45 (44–46)	47 (35–57)	0.18	0.18	1
MCS	49 (45–59)	51 (39–58)	55 (45–60)	0.27	**0.07**	**0.01**	50 (48–58)	48 (45–56)	54 (49–56)	0.8	0.8	0.67
MH	75 (61–90)	80 (55–85)	75 (70–90)	0.2	0.26	**0.05**	75 (72–88)	70 (60–80)	70 (68–85)	0.17	0.59	0.59
PCS	46 (44–53)	36 (29–39)	43 (37–47)	**0**	**0**	**0**	50 (42–51)	33 (32–34)	35 (29–43)	**0.02**	**0.02**	0.55
PF	85 (69–95)	50 (32–65)	75 (50–82)	**0**	**0**	**0**	80 (62–95)	40 (30–52)	65 (25–70)	**0.02**	**0.04**	0.55
RE	83 (75–100)	62 (31–83)	92 (58–100)	**0**	0.42	**0**	88 (75–100)	88 (56–100)	83 (69–98)	0.42	0.71	0.85
RP	56 (41–72)	25 (0–38)	50 (38–62)	**0**	**0.06**	**0**	50 (38–69)	25 (25–38)	44 (6–47)	**0.04**	0.21	0.89
SF	75 (62–100)	62 (44–88)	75 (62–100)	**0.03**	0.22	**0**	75 (62–94)	62 (56–81)	75 (75–88)	0.11	0.75	0.6
VT	62 (50–75)	44 (25–60)	62 (44–72)	**0**	0.64	**0**	62 (50–66)	38 (31–50)	56 (34–72)	**0.04**	0.29	0.6

*Comparison between baseline and 6-week and 4-month status. 0 = maximum disability, and 100 = no disability. Significant different results compared to baseline (pre) and 6 weeks were analyzed according to Paired Wilcoxon-signed rank test and highlighted in bold. PF, physical functioning; RP, role physical; BP, bodily pain; GH, global health; VT, vitality; SF, social functioning; RE, role emotional; MH, mental health; PCS, physical component summary; MCS, mental component summary*.

**Figure 3:**
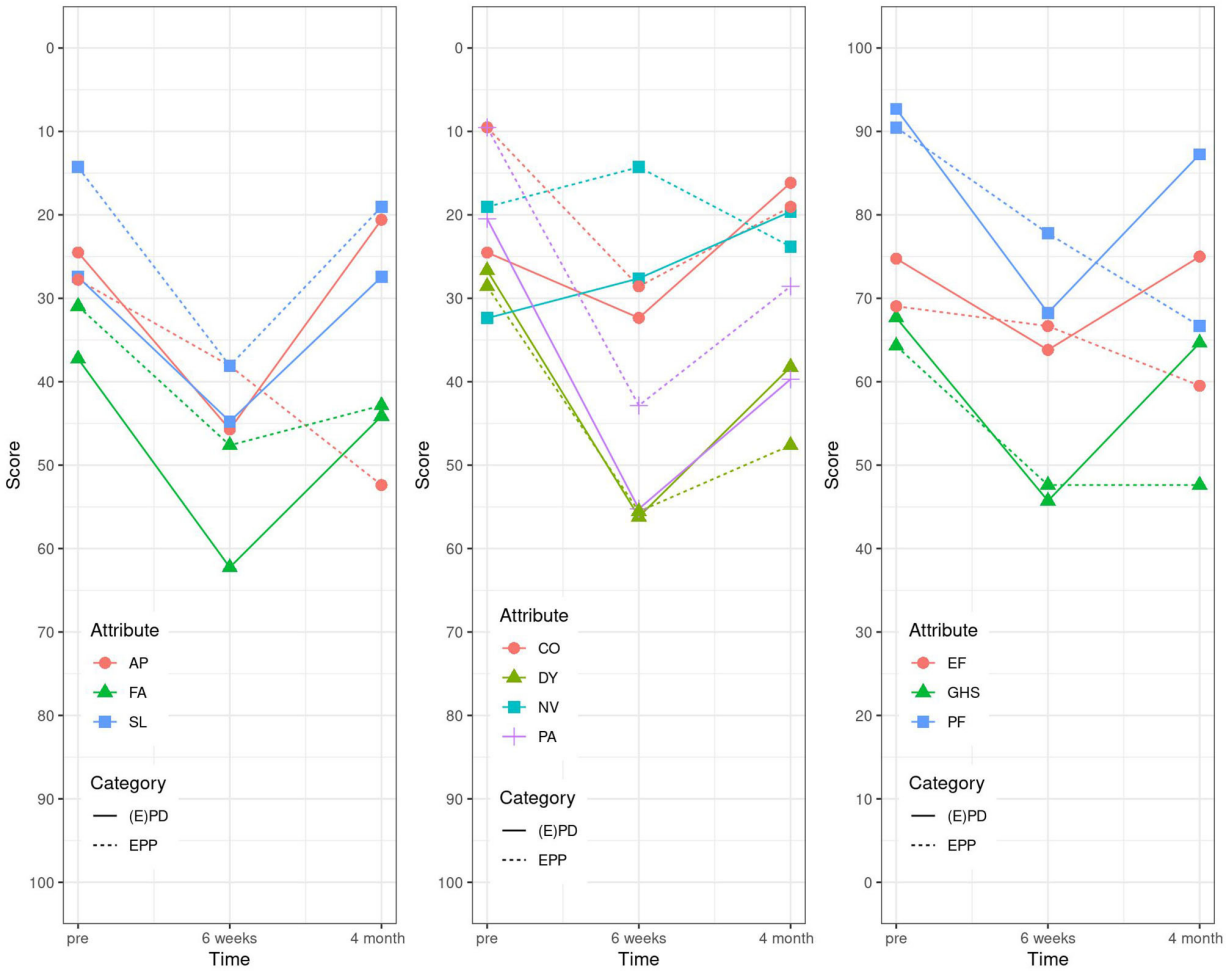
Linear graphs. EORTC C15 (2006): “not at all” corresponding to 0 and “very much” to 100. GHS, global health status; PF, physical functioning; EF, emotional functioning; FA, fatigue; NV, nausea and vomiting; PA, pain; DY, dyspnea; SL, insomnia; AP, appetite loss; CO, constipation.

The results of the EORTC C15 questionnaire in the (E)PD group showed that all values came back to baseline except for FA, PA, SL, and PF at 4 months after surgery, whereas in the EPP group, there was still an impairment seen for the items, PF, PA, and AP. An improvement compared with the baseline values was seen for PF in both groups (Table 3 and Figure 3).

**Table 3:** EORTC C15 (2006).

	**Pre**	**6 w**	**4 m**	***p* (6 w-pre)**	***p* (4 m-pre)**	***p* (4 m−6 w)**	**Pre**	**6 w**	**4 m**	***p* (6 w-pre)**	***p* (4 m-pre)**	***p* (4 m−6 w)**
	**EPD**						**EPP**					
AP	0 (0–33)	33 (0–83)	0 (0–33)	**0**	0.29	**0**	0 (0–50)	33 (17–50)	67 (17–83)	0.85	0.59	0.42
CO	0 (0–33)	33 (0–67)	0 (0–33)	0.27	0.19	**0.02**	0 (0–17)	33 (0–33)	0 (0–17)	0.34	1	0.78
DY	33 (0–33)	67 (33–100)	33 (33–67)	**0**	0.14	**0.01**	33 (0–50)	67 (42–67)	33 (33–67)	**0.09**	0.17	0.59
EF	83 (67–100)	67 (50–83)	83 (67–100)	**0.04**	0.53	**0.02**	67 (50–92)	67 (58–75)	67 (50–75)	0.85	0.75	0.79
FA	33 (17–50)	67 (50–83)	50 (33–67)	**0**	0.23	**0**	33 (8–50)	50 (42–50)	33 (25–58)	0.34	0.5	0.79
GHS	67 (67–83)	50 (33–50)	67 (67–67)	**0**	0.51	**0**	50 (50–75)	50 (42–50)	50 (33–67)	0.1	0.28	0.89
NV	33 (0–50)	33 (0–33)	0 (0–33)	0.55	0.18	0.18	0 (0–33)	0 (0–17)	0 (0–33)	0.85	1	0.35
PA	17 (0–33)	50 (33–83)	33 (33–67)	**0**	**0.01**	**0.01**	17 (0–17)	50 (33–58)	33 (0–33)	**0.03**	0.28	0.5
PF	100 (89–100)	67 (56–89)	89 (78–100)	**0**	0.11	**0**	89 (89–100)	78 (67–89)	78 (50–94)	0.05	0.17	0.58
SL	0 (0–58)	33 (17–67)	33 (0–33)	**0.09**	0.86	**0.02**	0 (0–33)	33 (33–50)	0 (0–33)	0.1	0.59	0.09

*Score of 0 = “not at all” and score of 100 = “very much.” Significant different results compared to baseline (pre) and 6 weeks were analyzed according to paired Wilcoxon-signed rank test and highlighted in bold. GHS, global health status; PF, physical functioning; EF, emotional functioning; FA, fatigue; NV, nausea and vomiting; PA, pain; DY, dyspnea; SL, insomnia; AP, appetite loss; CO, constipation*.

**Figure 4:**
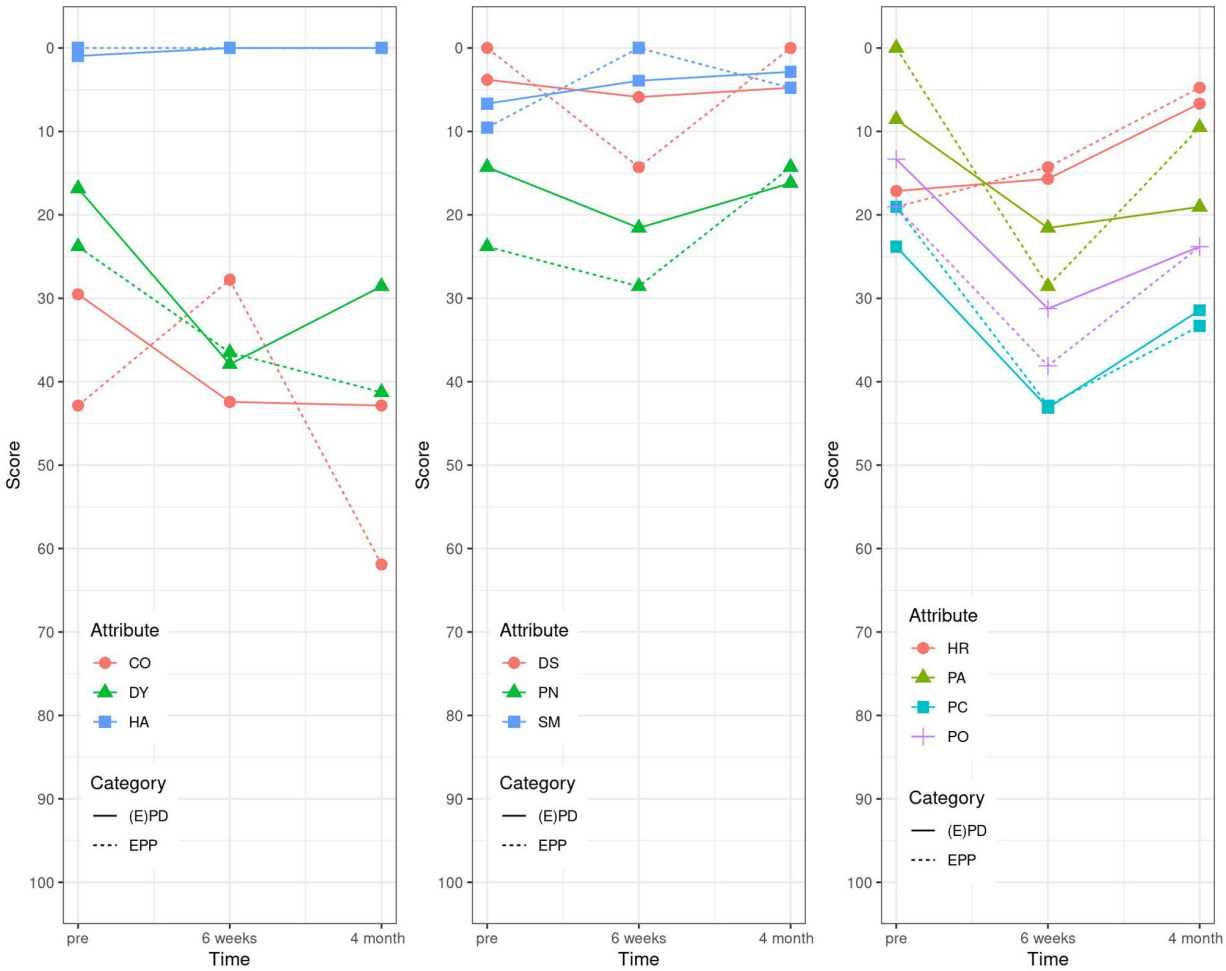
EORTC LC 13 (1994): high score for the scales and single items represents a high level of problems/symptomatology. DY, dyspnea; CO, coughing; HA, hemoptysis; SM, sore mouth; DS, dysphagia; PN, peripheral neuropathy; HR, alopecia; PC, pain in chest; PA, pain in arm/shoulder; PO, pain in other parts.

For the last EORTC LC 13 questionnaire, the items coughing (CO), dysphagia (DS), hemoptysis (HA), alopecia (HR), pain in arm/shoulder (PA), pain in chest (PC), peripheral neuropathy (PN), and sore mouth (SM) came all back to baseline values at already 6 weeks post-operatively in the EPD group, the same in the EPP group except for PA, DY, and pain in other parts (PO). PN was even better than at baseline (Table 4 and Figure 4).

**Table 4:** EORTC LC 13 (1994).

	**Pre**	**6 w**	**4 m**	***p* (6 w-pre)**	***p* (4 m-pre)**	***p* (4 m−6 w)**	**Pre**	**6 w**	**4 m**	***p* (6 w-pre)**	***p* (4 m-pre)**	***p* (4 m−6 w)**
	**EPD**						**EPP**					
CO	33 (0–33)	33 (33–67)	33 (33–67)	0.1	**0.01**	0.6	33 (33–50)	33 (8–33)	67 (50–83)	0.17	0.13	0.1
DS	0 (0–0)	0 (0–0)	0 (0–0)	0.43	0.77	0.61	0 (0–0)	0 (0–0)	0 (0–0)	1	NA	1
DY	11 (0–22)	33 (22–56)	22 (22–33)	**0**	**0**	**0.06**	22 (11–39)	44 (28–44)	22 (17–67)	**0.07**	0.17	0.79
HA	0 (0–0)	0 (0–0)	0 (0–0)	1	1	NA	0 (0–0)	0 (0–0)	0 (0–0)	NA	NA	NA
HR	0 (0–33)	0 (0–33)	0 (0–0)	0.71	**0.01**	**0.02**	0 (0–33)	0 (0–17)	0 (0–0)	1	0.37	1
PA	0 (0–0)	0 (0–33)	0 (0–33)	**0.01**	**0.04**	0.43	0 (0–0)	33 (17–33)	0 (0–17)	**0.05**	0.35	0.09
PC	33 (0–33)	33 (33–67)	33 (0–33)	**0**	0.4	**0.06**	33 (0–33)	33 (33–67)	33 (0–50)	0.1	0.41	0.59
PN	0 (0–33)	0 (0–33)	0 (0–33)	0.16	0.71	0.24	33 (0–33)	0 (0–50)	0 (0–17)	1	0.71	0.34
PO	0 (0–33)	17 (0–67)	0 (0–33)	**0.01**	0.1	0.09	0 (0–33)	33 (17–67)	33 (0–33)	0.18	1	0.5
SM	0 (0–0)	0 (0–0)	0 (0–0)	0.48	0.24	0.82	0 (0–0)	0 (0–0)	0 (0–0)	1	1	1

*0 = no symptoms, 100 = high symptom burden. DY, dyspnea; CO, coughing; HA, hemoptysis; SM, sore mouth; DS, dysphagia; PN, peripheral neuropathy; HR, alopecia; PC, pain in chest; PA, pain in arm/shoulder; PO, pain in other parts*.

**Figure 5:**
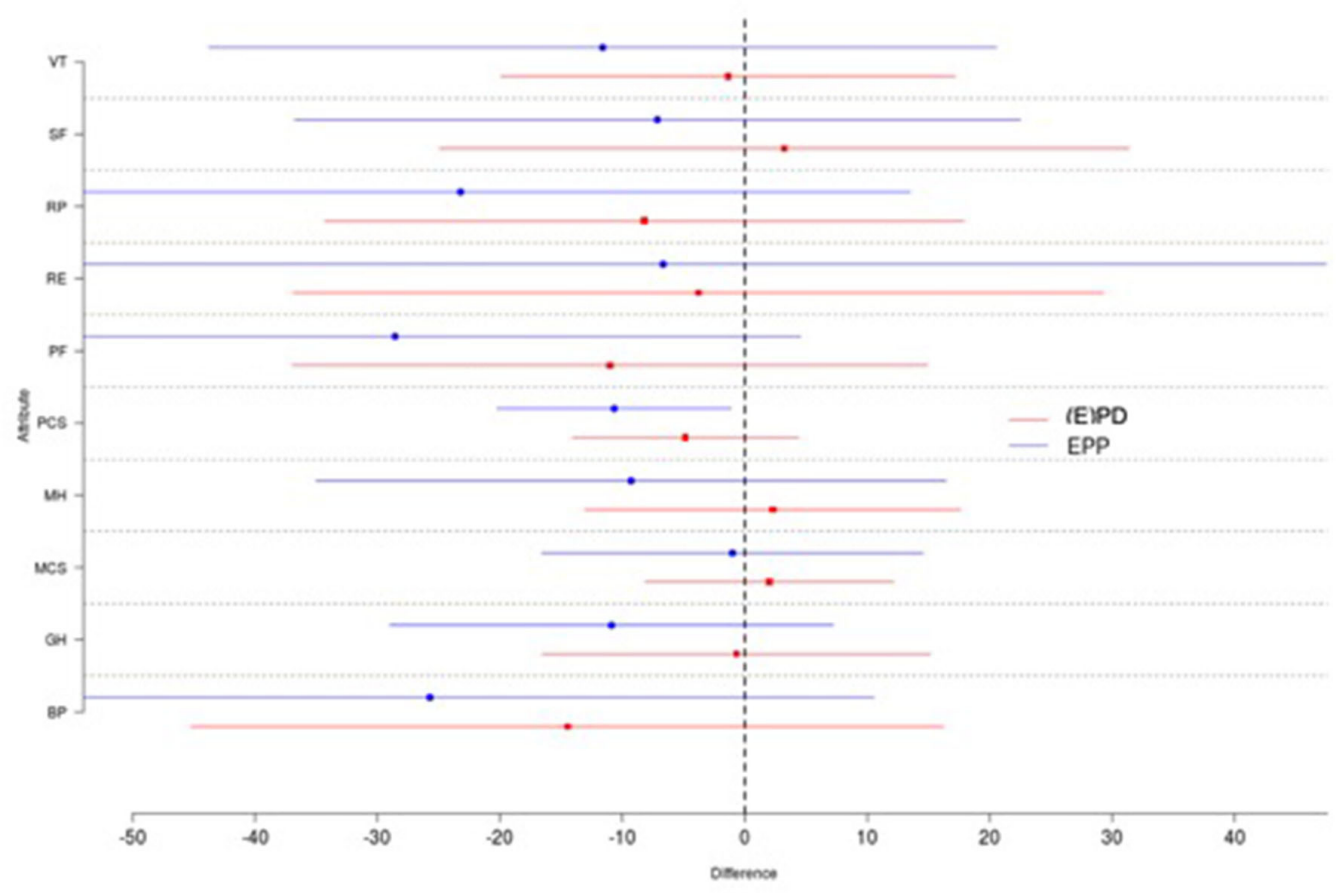
Shown are the means and standard deviations for each group and each attribute. One does not observe significant differences between the (E)PD (red) and EPP (blue) patients. There exists a trend toward a higher quality of life for the (E)PD treatment group as every single difference in the (E)PD group is further to the right compared to the EPP group. VT, vitality; SF, social functioning; RP, role physician; RE, role emotional; PF, physical functioning; PCS, physical component status; MH, mental health; MCS, mental component status; GH, global health; BP, bodily pain.

Further, the difference-in-difference analysis was performed to reduce confounding influence and confounding based on lose assumptions, as it might be the case that one group was doing better to begin with. Results of the difference-in-differences analysis for all attributes are summarized as shown in Figure 5. One does not observe significant differences between the patients with (E)PD and EPP. However, there was a trend toward a higher quality of life for the (E)PD treatment group compared to the EPP group in the SF-36 questionnaire. For the attributes social functioning (SF), mental health (MH), and mental component summary (MCS), an average increase of the values at 4 post-operative months compared to pre-operative values was observed in the (E)PD group, even if the difference was not statistically significant.

### Lung Function

Forced expiratory volume in 1 s showed a decrease after surgery in both groups: 76 vs. 48% in the EPP group and 82 vs. 65% in the (E)PD group with statistical significance for the (E)PD group with *p* < 0.001. FVC showed the same trend: 78 vs. 52% and 84 vs. 66%, respectively. Although the diffusion capacity did only significantly differ in the (E)PD group with a DLCO of 75 vs. 63% (*p* < 0.001), in the EPP group, DLCO was 73% pre-operatively and 55% post-operatively without statistical significance (Table 5).

**Table 5:** Pulmonary function test.

	**(E)PD**				**EPP**			
	**DLCO median (range)**	**FEV1**	**FVC**	**FEV1/FVC**	**DLCO**	**FEV1**	**FVC**	**FEV1/FVC**
Pre	75 (68–85)	82 (71–88)	84 (75–98)	77 (68–80)	73 (73–75)	76 (66–80)	78 (70–92)	70 (70–75)
Post (4 months)	63 (55–77)	65 (60–76)	66 (56–72)	81 (75–85)	55 (44–64)	48 (48–50)	52 (44–53)	74 (70–83)
*p* (post–pre)	**<0.001**	**<0.001**	**<0.001**	**<0.001**	0.1	0.06	0.06	0.1

*Pre-operative and 4 months post-operative lung function values for (E)PD and EPP*.

## Discussion

To our knowledge, this is the first analysis prospectively assessing QoL using three different questionnaires and additionally lung function of patients with MPM undergoing EPP or (E)PD. MPM is a life expectancy limiting disease. Even if treated within a multimodality therapy concept, the median overall survival lies around 11–24 months (11, 12). Preservation of QoL must be considered in the decision-making process before proposing a long and stressful treatment. The shift toward (E)PD, due to its less invasive nature, showed to achieve lower morbidity and mortality rates. As described in a review by Cao et al., mortality rates after (E)PD compared with EPP were 2.9 and 6.8%, respectively, and morbidity rates for (E)PD of 27.9% compared with 62.0% undergoing EPP (13).

However, in our cohort, the two surgical procedures (E)PD and EPP, seem to be comparable in terms of QoL at 4 months post-operatively. As mentioned above, the patients' individual expectancy of an “active” life after the surgery is an important aspect that deserves proper consideration during counseling and multidisciplinary meetings of the patients. This could greatly influence the decision in favor or against surgery. In general, patients define their quality of life by the remaining ability to exercise their common activities, for example, playing an instrument or doing sports with extreme pulmonary stress, after the surgery. For those adaptation of the patient to the “subnormal” values of post-operative FEV1, resembling the pulmonary function is important to achieve. In addition to the medical indications, the achievable post-operative QoL needs to be discussed on an individual base. Data of the current literature for QoL after surgery are fragmentary and very heterogeneous.

To date, there are only two studies, to our knowledge, directly comparing QoL after EPP and (E)PD (4, 5). There are other studies investigating QoL after either EPP or (E)PD, but direct comparison was made with non-surgical treatments (6). Some of these studies demonstrated a tendency toward a better QoL (all assessed with different scores) after lung sparing surgery compared with EPP, by looking at the procedure-related outcomes separately. In their work, Ploenes et al. (4) concluded that patients undergoing EPP have a greater impairment in post-operative lung function and, consequently, in QoL compared with patients undergoing (E)PD up to 12 months after the surgery. This might be related to the fact that with decortication of entrapped lungs, lung function, and therefore QoL can improve. Additionally, patients with EPP had reduced pulmonary function combined with worse dyspnea compared to (E)PD group, whereas cough and pain were equal within both groups. They concluded that a deterioration in post-operative lung function can lead to dyspnea and therefore influencing the patient's QoL. This can also be seen in our data, where FEV1 and FVC showed a greater improvement in the (E)PD group compared with the EPP group. Dyspnea showed an overall deterioration 6 weeks after the surgery, but nearly improved 4 months post-operatively in comparison with baseline values. The improvement of FEV1 and FVC 4 months post-operatively was greater in the (E)PD group compared with the EPP group, which can be reflected in a better QoL. Symptoms such as cough and dyspnea did not improve, but mostly returned to baseline values in both groups. These symptoms are mostly the leading symptoms and a disturbing factor for patients pre-operatively and assuming to have an impact of QoL.

The other study by Rena et al. (5) directly compared QoL in patients undergoing EPP (*n* = 40) and P/D (*n* = 37), at baseline, 6 and 12 months after the surgery by using EORTC-QLQ-C30 questionnaire. Both (E)PD and EPP caused a significant decrease within all variables at the 6 month mark. EPP had a worse long-term post-operative QoL compared with (E)PD. Only patients with P/D demonstrated a return to baseline values at 12 months post-operatively. This data favors P/D as the procedure with less limitation in patient's QoL.

When compared with the above-mentioned results of the two studies with our patient cohort, our data demonstrate, in the majority of items, a closer approach to baseline values in the (E)PD group compared with the EPP group, even though a tendency returning to pre-operative status was documented at 4 months after the operation for both surgical groups. The better overall improvement in the (E)PD group reflects the less invasive nature.

Besides the procedure itself, patient selection and pre-operative patient characteristics play a crucial role for the peri- and post-operative morbidity and mortality. The ASA II classification was greater represented in the EPD group with 60% than with 14% in the EPP group. ASA III was 37 and 86%, respectively, although without statistical significance. The post-operative morbidity rate additionally influences QoL, arises, among other things, from prolonged air leak, which more often occurs in the (E)PD group than in the EPP group, leading to a longer hospital stay, also implicates the importance of an accurate patient selection. Deciding which patients are suitable for the surgery and which are not suitable remains the main challenge, even for high volume and experienced centers.

Within questionnaires, the items role emotional, mental status, and physical functioning showed an improvement post-operatively. This may implicate that patient experience a positive psychological effect due to the tumor resection. This partly holds on up to 4 months post-operatively and was equal in both groups, although patients with (E)PD even had a longer recovery time in most of the cases, which is supposed to influence the patient's mental status. These results may implicate a favor of (E)PD and ultimately are the preferred procedure.

Even though for most of the above-mentioned symptoms, the improvement did not reach a statistical significance, their impact on QoL, in our opinion, is beyond any doubt. The results show a clear trend in favor of (E)PD for almost every single attribute indicating that this surgical approach is associated with less deterioration in QoL and therefore should be the favored approach, whenever feasible.

## Limitation

One of the main limitations of this study may be the short post-operative observation time. In our institution, we experienced the greatest improvements within the immediate post-operative time and therefore decided to limit the follow-up to 4 months post-operatively. Another reason is that some patients will be followed-up in a hospital closer to their hometown afterward and may get lost at follow-up. The missing statistically significant difference in quality of life of patients with (E)PD compared with patients with (E)PP was probably due to the smaller sample size in the latter group. Therefore, these data are rather indicative. We did not adjust for confounders, and there are no causal relationships; yet these results show a clear trend, although the results must be interpreted with caution.

## Conclusion

The present analysis represents a correlative study not a causal analysis. QoL after EPP in comparison with (E)PD, according to our data did not show an impairment and therefore EPP should not be abandoned for QoL reasons, if medically indicated. On the contrary, the decision for (E)PD should not only relay on minor morbidity and mortality rates, but also on improved QoL, at least for certain items, as demonstrated in our data. In summary, the small sample size does not allow us to draw any causal correlation.

## Data Availability Statement

The raw data supporting the conclusions of this article will be made available by the authors, without undue reservation.

## Ethics Statement

The studies involving human participants were reviewed and approved by Kantonale Ethikkommission Zürich (StV 29-2009, EK-ZH 2012-0094). The patients/participants provided their written informed consent to participate in this study.

## Author Contributions

OL wrote, reviewed and edited the manuscript, and she worked on the methodology, the validation of the data, and the visualization. MP was a contributor in the writing, reviewing, and editing of the manuscript. TN performed the statistical analysis. II and WW reviewed the manuscript. IO came up with the concept and designed the work, and she was a major contributor in the writing, reviewing and editing of the manuscript, worked on the methodology, and supervised the project. All authors contributed to the article and approved the submitted version.

## Funding

Work on this project was in part funded by Swiss National Science Foundation (PP00P3_159269 and PP00P3_133657).

## Conflict of Interest

IO holds an advisory board role for AstraZeneca and MSD, has received speakers fee from Roche and AstraZeneca, and institutional research grants from Medtronic and Roche. The remaining authors declare that the research was conducted in the absence of any commercial or financial relationships that could be construed as a potential conflict of interest.

## Publisher's Note

All claims expressed in this article are solely those of the authors and do not necessarily represent those of their affiliated organizations, or those of the publisher, the editors and the reviewers. Any product that may be evaluated in this article, or claim that may be made by its manufacturer, is not guaranteed or endorsed by the publisher.

## Abbreviations

AP: appetite loss
BP: bodily pain
CO: constipation
CO: coughing
DLCO: diffusing capacity of the lung for carbon monoxide
DS: dysphagia
DY: dyspnea
EF: emotional functioning
EORTC: European Organization for Research and Treatment of Cancer
(E)PD: extended pleurectomy/decortication
EPP: extrapleural pneumonectomy
FA: fatigue
FEV1: forced expiratory volume in 1 s
FVC: forced volume vital capacity
GH: global health
GHS: global health status
HA: hemoptysis
HR: alopecia
IQR: interquartile range
MCR: macroscopic complete resection
MCS: mental component summary
MH: mental health
MPM: malignant pleural mesothelioma
NV: nausea and vomiting
OS: overall survival
PA: pain
PA: pain in arm/shoulder
PC: pain in chest
PCS: physical component summary
P/D: pleurectomy/decortication
PO: pain in other parts
PFS: progression-free survival
PF: physical functioning
PN: peripheral neuropathy
RE: role emotional
RP: role physical
SF: social functioning
SL: insomnia
SM: sore mouth
QoL: quality of life
VT: vitality.
